# Maternal Cardiovascular Outcomes of Pregnancy in Childhood, Adolescent, and Young Adult Cancer Survivors

**DOI:** 10.3390/jcdd9110373

**Published:** 2022-10-31

**Authors:** Neha Bansal, Carol Fernandez Hazim, Sergio Badillo, Sharvari Shyam, Diana Wolfe, Anna E. Bortnick, Mario J. Garcia, Carols J. Rodriguez, Lili Zhang

**Affiliations:** 1 The Children’s Heart Center, Kravis Children’s Hospital, Icahn School of Medicine at Mount Sinai, New York, NY 10029, USA; 2 Department of Medicine, Montefiore Medical Center, Bronx, New York, NY 10467, USA; 3 Cardiology, CEDIMAT Cardiovascular Center, Santo Domingo 10514, Dominican Republic; 4 Department of Pediatrics, St. Barnabas Hospital, Bronx, New York, NY 10457, USA; 5 Department of Obstetrics & Gynecology, Montefiore Medical Center, Albert Einstein College of Medicine, Bronx, New York, NY 10461, USA; 6 Women’s Health, MFM-Cardiology Joint Program, Montefiore Medical Center, Albert Einstein College of Medicine, Bronx, New York, NY 10461, USA; 7 Department of Medicine, Division of Geriatrics, Montefiore Medical Center, Albert Einstein College of Medicine, Bronx, New York, NY 10461, USA; 8 Department of Medicine, Division of Cardiology, Montefiore Medical Center, Albert Einstein College of Medicine, 111 E 210th St, Bronx, New York, NY 10467, USA

**Keywords:** cancer survivors, cardiotoxicity cardiovascular outcomes, pregnancy

## Abstract

This review focuses on the maternal cardiovascular risk and outcomes of pregnancy in childhood, adolescent, and young adult cancer survivors who are achieving survival to their prime reproductive years. Childhood, adolescent, and young adult cancer survivors are a growing population and have increasing needs for reproductive care over decades of life. Female cancer survivors have an overall higher risk of maternal cardiovascular events compared to those without a history of cancer. In female cancer survivors with normal cardiac function before pregnancy, the incidence of new heart failure during pregnancy is low. In survivors with cardiotoxicity prior to pregnancy, the risk of heart failure during and immediately after pregnancy is much higher. We recommend cardiomyopathy surveillance with echocardiography before pregnancy for all female survivors treated with anthracyclines and chest radiation. Survivors with cardiotoxicity prior to pregnancy should be cared for by an expert multidisciplinary team, including obstetrics, cardiology, anesthesia, and specialized nursing, among others.

## 1. Introduction

This review focuses on the maternal cardiovascular risk and outcomes of pregnancy in childhood, adolescent, and young adult cancer survivors who are achieving survival to their prime reproductive years. Childhood, adolescent, and young adult cancer survivors are a growing population, and concerns about reproductive potential should be anticipated as they mature. Cardiovascular disease is a prominent co-morbidity among survivors of malignancies [1]. The most common cardiac diagnoses in cancer survivors are congestive heart failure, myocardial infarction, pericardial disease, and valvular dysfunction [2]. Young adolescent women with cancer have an increased risk of primordial ovarian insufficiency [2] due to treatment with high-dose irradiation or cytotoxic chemotherapy. Yet, many remain fertile and desire pregnancy [3]. Thus, cardiologists, oncologists, and obstetricians are integral to the care of women cancer survivors of reproductive age.

**Figure 1:**
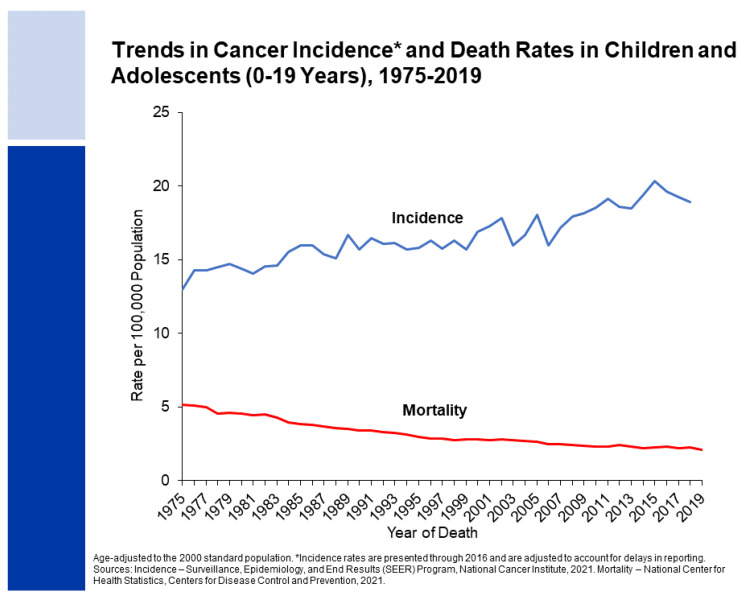
Cancer incidence rates and mortality rates for children and adolescents, from 1975 through 2019 [6].

Cancer is one of the leading causes of morbidity and mortality in children and adolescents [4]. In 2022, approximately 10,470 children (birth to 14 years) and 5480 adolescents (aged 15–19 years) will be diagnosed with cancer, and 1050 and 550, respectively, will die from the disease [5]. Nearly 70,000 adolescents and young adults between the ages of 15 and 39 years are diagnosed with cancer annually in the United States [1]. Leukemia is the most common childhood cancer, accounting for 28% of cases, followed by brain and other nervous system tumors (27%) [4]. The 5-year survival rate for all childhood cancers combined improved from 58% during the mid-1970s to 85% from 2011 through 2017 in children and from 68% to 86% in adolescents [6]. Since 1975, cancer incidence rates have increased slightly by about 0.8% per year, whereas mortality rates have declined by 71% and 61% for children and adolescents, respectively, from 1970 through 2019 (Figure 1). The large mortality reduction is largely because of improved treatment for leukemia. While in childhood (ages 0–14 years), the incidence of cancer combined is about 10% higher in boys than in girls (incidence rate ratio 1.11; 95% confidence interval (CI) 1.09–1.13), it is equal between 15 to 19 years [7,8]. The incidence of cancer is 30% higher in women between 20 to 29 years and is double in women as compared to men between 30 to 39 years, largely because of breast cancer [8]. Thus, childhood, adolescent, and young adult cancer survivors have growing needs for reproductive care over decades of life.

## 2. Type of Cardiotoxic Cancer Therapies

Anthracyclines are the chemotherapeutic drug class of choice for treating many cancers [9]. They are the most used chemotherapeutic agents in pediatrics, used in almost all pediatric cancer regimens (10). Anthracyclines have been the mainstay of adjuvant chemotherapy agents for breast cancer in young women. Clinically significant cardiotoxicity is a major limitation of these medications (Table 1), and this has now led to the use of lower doses in cancer treatment [10,11]. Anthracyclines affect cardiac function mainly through mechanisms that involve reactive oxygen species formation, induction of apoptosis, DNA damage through interaction with topoisomerase II, and inhibition of protein synthesis [12]. They exert their anti-cancer effect by either intercalating between base pairs of DNA, preventing malignant cell replication, or by inhibiting topoisomerase II activity that then prevents the uncoiling process of DNA required for replication [13]. Anthracyclines target topoisomerase II, which relieves the stress on DNA when unwinding, by causing breaks and then resealing the DNA [14]. Anthracycline cardiotoxicity was categorized at the time of presentation as either acute (during or within 2 weeks after completing therapy) or chronic, with chronic cases further categorized as early (within 1 year after completing therapy) or late onset (>1 year after completing therapy) [15]. Acute symptoms can occur within hours of infusion and may include arrhythmias, heart failure, myocarditis, and pericarditis [16]. These symptoms may resolve after stopping the treatment, but cardiac function may decline with time. Chronic symptoms can present months to years post-therapy, as the decreased thickness of the left ventricular (LV) wall results in a decline in cardiac function, eventually progressing to heart failure (HF) [16]. Anthracycline cardiotoxicity is a continuous phenomenon, from myocardial cell damage, followed by a subclinical functional decline, to clinically manifest HF [17]. Cardiotoxicity is dose-dependent and there is no safe dose but there is clearly an increased risk of cardiomyopathy beyond 250 mg/m^2^ [2,18].

Cardiac radiation exposure can increase the risk of HF (Table 1), particularly beyond 15 Gy of total chest radiation doses, and higher yet at doses exceeding 35 Gy [2]. Below 15 Gy is associated with a lower risk of HF and no screening is recommended [20]. Radiation can lead to the formation of free radicals, generation of reactive oxygen species, cytokine release, and endothelial injury. This inflammatory process can further lead to atherosclerosis through ruptured vessel walls, platelet aggregation, thrombosis, and replacement of the damaged coronary intima cells by myofibroblasts. With radiation, fibrosis can develop at the valves, myocardium, and epicardium leading to valvular heart disease, cardiomyopathy, and pericarditis. The conduction system can be directly injured by radiation through an inflammatory process resulting in fibrosis or indirectly via fibrosis after ischemia of the myocardium [21]. The Childhood Cancer Survivor Study Cohort (CCSS) demonstrated that childhood Hodgkin’s lymphoma survivors regardless of therapy had increased risks compared to siblings for HF, myocardial infarction, pericardial disease, and valvular dysfunction [2]. Most radiation-induced cardiovascular events become apparent after decades of follow-up [22]. A study of 2617 five-year survivors of Hodgkin lymphoma showed that the risk of HF was further increased in patients treated with anthracyclines [23]. Radiation-induced pericarditis occasionally leads to constrictive pericarditis, which may be life-threatening. However, recently, this has become rare due to advances in radiation protocols (improved techniques, lower dosages, and less volume exposed) [24]. In contrast, chronic pericarditis remains a frequent radiation-induced cardiotoxic effect [22].

Human epidermal growth factor receptor 2 (HER2)-targeted therapy, i.e., trastuzumab, a class of medicines used to treat all stages of HER2-positive breast cancer, has also been associated with cardiotoxicity (Table 1). Opposite to anthracyclines that directly cause structural damage to cardiomyocytes, its mechanisms of action include cytotoxicity through inhibition of signal transduction, neoangiogenesis, and repair of DNA damage caused by other treatments [25]. Risk factors for cardiotoxicity include previous or concomitant anthracycline treatment, short time between anthracyclines and HER2-targeted treatment, age >65 years, high body mass index (>30 kg/m^2^), previous LV dysfunction, arterial hypertension, and previous radiation therapy [25]. In a recent meta-analysis of 18,111 patients from 6 randomized control trials, the overall incidence of high-grade HF in patients treated with trastuzumab versus placebo was 1.4% and the relative risk of cardiotoxicity increased with the duration of trastuzumab treatment. In a nationwide Danish cohort study, trastuzumab treatment after anthracycline-based chemotherapy was associated with a 2-fold increased risk of late clinical HF compared to that after anthracycline-based chemotherapy alone [26].

Tyrosine kinase inhibitors (TKI) are novel therapies that have revolutionized the treatment of many cancers. Inhibition of the vascular endothelial growth factor (VEGF) signaling pathway (VSP) is responsible for preventing tumor angiogenesis and has been associated with various cardiovascular toxicities including cardiomyopathy, hypertension, arterial and/or venous thrombosis, and renal vascular injury [27]. The overall incidence of hypertension ranges from 20 to 25% with bevacizumab and sunitinib (the initially approved drugs in this class) to >50% with newer agents [28]. The use of bevacizumab in a total of 16,962 patients from 19 randomized controlled trials was significantly associated with an increased risk of high-grade HF in cancer patients with a relative risk of 1.98 [29].

Studies have shown that some non-anthracycline chemotherapy drugs are associated with cardiotoxic effects as well (Table 1) [30]. Drugs such as alkylating agents (e.g., cyclophosphamide), microtubule inhibitors, proteasome inhibitors, platinum-based drugs, and antimetabolites are associated with ventricular dysfunction, myocardium ischemia, venous thromboembolism, arrhythmia, and QT prolongation [30]. More importantly, these therapies are often used in combination or sequentially, which can further enhance the risk of cardiovascular disease. Hence, screening for cardiotoxicity and early intervention is exceedingly important in pregnant cancer survivors.

Echocardiography plays a critical role in the assessment of cardiotoxicity. It provides a comprehensive evaluation of left ventricular ejection fraction (LVEF), valvular and pericardial evaluation, and hemodynamic examination. Multiple guidelines have recommended echocardiographic evaluation of LVEF (ideally the three-dimensional, but at least the two-dimensional Simpson biplane method) at the initiation of cardiotoxic treatment, during active cardiotoxic treatment and many years into survivorship to monitor the occurrence of LV dysfunction [31,32,33,34,35]. Global longitudinal strain (GLS) by speckle tracking echocardiography has also been recommended as a sensitive marker to detect subclinical LV dysfunction and has become the standard of care in cardio-oncology practice [31,32,33,34,35]. In general, overt cancer treatment-related LV dysfunction is defined as an absolute LVEF drop of >10% to a value <50% or an LVEF drop of >20%. A relative drop of GLS ≥12–15% from baseline is also considered a decline of clinical significance [31,32,33,34,35]. Other imaging modalities, such as radionuclide angiography or cardiac magnetic resonance imaging, may be considered for the evaluation of cardiotoxicity in at-risk survivors for whom echocardiography is not optimal. In the pediatric population, although a recent meta-analysis revealed that myocardial strain imaging unveils early evidence of myocardial injury in children with cancer and long-term survivors, data are unclear in the clinical adaptation of this finding [36]. There are no data to support the alteration of chemotherapeutic regimens on the basis of LV GLS in pediatrics. However, some data suggest that the use of certain cardioprotective strategies, such as using angiotensin-converting enzyme inhibitors (ACEIs), may help with improvement in GLS [37]. However, whether this translates into the prevention of cardiac outcomes is unknown.

## 3. Cardioprotective Strategies

Dexrazoxane works by chelating iron and interfering with iron-mediated free radical generation, ultimately decreasing tissue damage caused by anthracyclines [12,38]. It is a primary cardioprotectant agent approved by the United States Food and Drug Administration to prevent anthracycline cardiotoxicity [39]. It is important to note that dexrazoxane does not decrease the efficacy of cancer treatment, nor does it compromise event-free survival [38]. The Dana Farber Cancer Institute’s Childhood Acute Lymphoblastic Leukemia Consortium and the Children’s Oncology Group currently include dexrazoxane in their research protocols that involve anthracyclines [40]. Cancer survivors should be encouraged to exercise regularly to improve exercise capacity, weight, mental status, and cardiometabolic risk as a cardioprotective strategy, as cancer survivors have shown to have poor cardiorespiratory fitness with reduced peak oxygen uptake (VO2 max) during exercise testing [41]. Long-term survivors of childhood cancer also have an increased incidence of dyslipidemia, hypercholesterolemia, and hypertriglyceridemia [42,43]. Although theoretically, statins may be cardioprotective in patients with several cardiovascular risk factors, the benefits and risks remain unclear. There are ongoing prospective randomized studies examining the cardioprotective effect of statin therapy in patients undergoing anthracycline-based chemotherapy (Preventing Anthracycline Cardiovascular Toxicity With Statins (PREVENT) study (ClinicalTrials.gov: NCT01988571) and Statins TO Prevent the Cardiotoxicity From Anthracyclines (STOP-CA) study (ClinicalTrials.gov: NCT02943590)). Guideline-directed medical therapy is often used for chemotherapy-induced cardiac dysfunction and symptomatic heart failure, in both children and adults. A few studies exist with mixed outcomes for the use of beta-blockers. In the Carvedilol for Prevention of Chemotherapy-Related Cardiotoxicity (CECCY) trial, carvedilol had no impact on the incidence of ≥10% LVEF reduction within 6 months. However, the use of carvedilol resulted in a significant reduction in troponin levels and diastolic dysfunction. [44]. In another placebo-controlled clinical trial of carvedilol, the mean LVEF declined in the placebo group in 6 months but was maintained in the carvedilol group (*p* < 0.001) [45]. Additionally, women with breast cancer receiving carvedilol vs. placebo revealed that strain and strain-rate measurements in the women receiving carvedilol were closer to normal than in women receiving placebo, but mean LVEF did not differ significantly between groups [46]. Similar mixed studies exist for the use of ACEIs or angiotensin II receptor blockers (ARBs) in this population. A randomized study of enalapril vs. placebo found higher troponin concentrations in the placebo group but found no difference in mean LVEF at 6 months [47]. The OVERCOME Trial showed that patients treated with enalapril and carvedilol had smaller reductions in LVEF than those in untreated controls during the 6 months of observation [48]. A meta-analysis concluded that the angiotensin antagonists (*p* < 0.001) and beta-blockers (*p* < 0.001) prevented chemotherapy-induced cardiotoxicity in the short term [49]. However, it is unclear that if changes in LVEF actually translate into improved long-term outcomes, such as prevention of symptomatic HF or cardiovascular death in these survivors. It is of note that none of these studies included pregnant patients, as some of these medications, such as ACEIs, are contraindicated during pregnancy.

Limited evidence suggests the use of alternative medicine to prevent cardiotoxicity. Ascorbic acid may reduce oxidative stress and may be considered in survivors of childhood cancer [50,51]. Some preclinical data suggest that there may be benefits of mycotherapy on tumor response, host immune functions, and inflammation in cancer patients [52]. In addition, there are studies on curcumin products with potential antitumor capabilities via affecting molecular pathways and may be considered in the prevention of adverse effects of doxorubicin on normal cells and tissues via reducing inflammation, oxidative stress, and apoptosis [53,54]. However, there have been no randomized control trials supporting the use of alternative medicine in clinical practice to prevent cardiotoxicity.

## 4. Maternal Cardiovascular Physiology of Pregnancy

During pregnancy, the cardiovascular system undergoes important structural and hemodynamic changes [55]. Major adaptations in pregnancy include vasodilation of the maternal systemic vasculature and kidneys, an increase in cardiac output, and a decrease in blood pressure, which mostly occurs during the first and second trimesters. Along with the change in cardiac output and pressure, there is a redistribution of blood flow to the uterus and placenta and significant increases in total blood volume, plasma volume, and red blood cell mass during pregnancy. Plasma volume increases more than the red blood cell mass, resulting in a “physiological anemia” due to the hemodilution [55].

During labor and delivery, the maximum cardiac output occurs due to increases in circulating catecholamines, preload, and heart rate, releasing 300 to 500 mL of blood into the systemic circulation with each uterine contraction [55]. Cardiac output can increase 60% to 80% postpartum compared to pre-labor values. These changes begin to resolve within the first 48 h up to the first two weeks postpartum; in some rare cases, they can last up to 6 months [55].

## 5. Maternal Cardiovascular Outcomes of Pregnancy in Cancer Survivors

### 5.1. Cardiomyopathy and Heart Failure

Chemotherapy-induced cardiomyopathy is a widely recognized complication of cancer therapies and has been defined as a decline in the LVEF and/or manifestations of symptomatic HF. Clinical HF has been reported in about 6% of patients undergoing anthracycline therapy, whereas subclinical LV dysfunction develops in 15–18% of patients [32,34,56,57]. In a recent prospective study on 2625 cancer patients who received anthracyclines, the incidence of anthracycline cardiotoxicity (LVEF decrease >10 absolute points, and <50%) was 9%; importantly, cardiotoxicity in 98% of the sample developed within the first year [57]. In childhood cancer survivors, female sex and the cumulative dose of anthracyclines are associated with an increased risk of cardiomyopathy [2,16,58,59].

Although a past cancer diagnosis is associated with an overall higher risk of maternal cardiovascular events compared to general pregnancy, the occurrence of new-onset cardiomyopathy during pregnancy in cancer survivors is low. In a recent population-based study of 4062 female cancer survivors of cancer diagnosed ≤21 years of age in Canada, survivors had a higher relative risk of cardiac morbidity during pregnancy (defined as any HF, arrhythmias, valvular disease, pericardial disease, coronary artery disease, or cardiac-related death) (relative risk = 4.18, 95% CI 1.89, 9.24), compared to matched controls without prior cancer [60]. In a meta-analysis of 6 studies consisting of 2016 pregnancies, predominantly in childhood cancer survivors, the weighted incidence of LV dysfunction or HF in those without a history of cancer therapy-related cardiac dysfunction was 0.24% [61]. In a retrospective cohort study of 847 female cancer survivors with 1554 completed pregnancies treated with anthracyclines, only 3 (0.3%) developed pregnancy-associated cardiomyopathy (defined as fractional shortening <28%, LVEF < 50%, or treatment for HF during pregnancy or within 5 months postpartum) [62]. In a smaller study, 29 women with a history of childhood cancer treated with anthracyclines had fractional shortening ≥30% at baseline and had no change in cardiac function during pregnancy [63]. Another study identified 94 pregnancies in 78 cancer survivors previously exposed to potentially cardiotoxic treatments (chemotherapy and/or radiation therapy to the thorax, including 55 women who received anthracyclines). The incidence of pregnancy-related HF (defined as LVEF <50% with or without HF symptoms) was 0% in survivors without prior cardiotoxicity [64]. These data support that in cancer survivors with normal cardiac function before pregnancy, the occurrence of new-onset HF during or immediately after pregnancy is low.

However, for childhood, adolescent, and young adult cancer survivors with preexisting chemotherapy-induced cardiomyopathy before pregnancy, their cardiovascular events rates appear to be higher. In a small prospective cohort study, 8 of 37 (22%) women with cardiomyopathy prior to pregnancy (fractional shortening of <30% before pregnancy), had a mean decrease in fractional shortening by 19% after pregnancy [63]. In a single-center study of 847 female cancer survivors with 1554 completed pregnancies, 26 women had a diagnosis of cardiac dysfunction prior to pregnancy, of which 8 had recurrent HF or worsened cardiac function during pregnancy [62]. Liu et al. reported the incidence of HF (defined as LVEF <50% with or without HF symptoms) during pregnancy and up to 16 weeks postpartum was as high as 31% in women with a history of cardiotoxicity, vs. none in those without a history of cardiotoxicity [64]. In a meta-analysis of 2016 pregnancies, the incidence of LV dysfunction or HF with pregnancy in survivors with a history of cancer therapy-related cardiac dysfunction was 28.4% (odds ratio of 47.4) in comparison to those without a history of cardiotoxicity prior to pregnancy [61]. These findings suggest an increased risk of cardiac function deterioration with pregnancy in survivors with previous cardiac dysfunction. Female cancer survivors with cardiotoxicity prior to pregnancy are recommended to have close cardiac surveillance during pregnancy at a center with expertise in cardiac disease in pregnancy [64].

### 5.2. Preeclampsia/Eclampsia

Preeclampsia is defined as new-onset hypertension and end-organ damage, including proteinuria, after 20 weeks of gestation, and it remains a leading cause of maternal and fetal morbidity and mortality [65]. Reports of preeclampsia and eclampsia in cancer survivors are sparse. Several cohort studies reported an approximately 5% absolute risk of preeclampsia during pregnancy in cancer survivors, and the rates were not higher or only modestly (1.4-fold) higher than in non-cancer controls [66,67]. In the British Childhood Cancer Survivor Study, a total of 2783 singleton pregnancies among 1712 female survivors of childhood cancer were investigated and survivors of Wilms tumor treated with abdominal radiotherapy were at a three-fold risk for the development of hypertension during pregnancy [68]. According to the National Inpatient Sample data in 2010–2014, pregnant cancer survivors had higher odds of preeclampsia (adjusted Odds Ratio 1.18, 95% CI 1.02, 1.36) [69].

### 5.3. Coronary Artery Disease

Coronary artery disease (CAD) is a complication of thoracic radiation. Studies have shown that Hodgkin lymphoma survivors (81% treated with mediastinal radiotherapy) had a 3.2-fold increased relative risk of CAD when compared with the general population, corresponding to an excess of 70 cases CAD per 10,000 person-years, and the highest relative risks were seen in patients treated before 25 years of age [70]. In women with breast cancer, rates of major coronary events increased linearly by 7.4%/Gy with the mean heart dose within the first 5 years after exposure and continued for at least 20 years [71]. Other cancer therapies associated with increased risk of CAD include fluorouracil, platinum drugs, and immune checkpoint inhibitors. The mechanism is likely through plaque formation and rupture, among others [72].

No literature or data exist on cancer survivors with CAD in the context of pregnancy. Women of reproductive age have a low risk of CAD; however, pregnancy is associated with a 3–4 fold increased risk of acute myocardial infarction [73]. In non-pregnant cancer survivors, the outcomes of acute coronary syndrome are worse when compared to patients without cancer [74].

### 5.4. Arrhythmias

Arrhythmias are one of the most common cardiac complications during pregnancy. Hormonal, autonomic, and hemodynamic changes contribute to arrhythmogenesis [75]. The prevalence of premature atrial contractions and premature ventricular contractions is high during pregnancy and supraventricular tachycardia is the most common sustained arrhythmia encountered during pregnancy, with a prevalence of 24 per 100,000 pregnancies [76]. Atrial fibrillation or flutter and ventricular tachycardia or fibrillation are rare during pregnancy, with a prevalence of 2 in 100,000 pregnancies [76].

There are no reports of arrhythmias during pregnancy in cancer survivors. However, it is known that anthracyclines can cause primary or secondary arrhythmias related to cardiomyopathy due to QT prolongation. Radiation therapy can cause myocardial fibrosis, which can provoke an arrhythmogenic focus. Fradley et al. showed that the rates and risk of arrhythmias in cancer survivors with chemotherapy-induced cardiomyopathy were similar to that of patients with other forms of non-ischemic cardiomyopathy [77].

### 5.5. Pericardial Disease

Mediastinal radiation is associated with a risk of acute pericarditis, chronic pericarditis, constrictive pericarditis, and effusive constrictive disease [21]. In 34,825 women treated with radiotherapy for breast cancer, the incidence ratio of pericarditis was 1.61 (95% CI 1.06, 2.43) in irradiated women with left-sided versus right-sided tumors [78]. The most common form of pericardial involvement during pregnancy is isolated pericardial effusion, followed by acute pericarditis, usually idiopathic. The general management of these conditions during pregnancy is not different from those of non-pregnant women. Breast cancer is the most common cause of malignant pericardial disease during pregnancy [79]. Khabele et al. reported a 28-year-old female who presented with cardiac tamponade during pregnancy as the first unusual presentation of advanced breast cancer [80].

Constrictive pericarditis during pregnancy is uncommon. There have been sparse case reports on pregnancy with constrictive pericarditis in non-cancer survivors [81] and cancer survivors [82]. It is thought that pregnant women who are in a satisfactory clinical state at baseline can tolerate pregnancy and labor without any serious problems [82].

### 5.6. Valvular Heart Disease

Chest radiation is a significant risk factor for valvular heart disease (19). The prevalence of radiation-induced valvular heart disease is around 16–39% at 10 years posttreatment and 16–60% at 20 years posttreatment, with aortic insufficiency and mitral regurgitation being the most common presentations [83]. A study of 1852 5-year survivors of Hodgkin’s lymphoma showed that 4.8% of survivors had valvular heart disease of at least moderate severity, most commonly affecting the aortic and/or mitral valves [84].

During pregnancy, stenotic lesions carry a higher risk of morbidity. Women with significant aortic or mitral stenosis should be carefully evaluated before conception to determine whether pre-pregnancy intervention warrants consideration [85]. Regurgitant lesions are often better tolerated due to the reduced systemic vascular resistance during normal pregnancy. However, in the early postpartum period, women with significant regurgitation may be at risk for pulmonary edema due to increased systemic vascular resistance after placental delivery, elevated volume returning to the circulation from the uterus, and preeclampsia [85].

### 5.7. Pulmonary Hypertension

Pulmonary hypertension can occur in cancer survivors either due to direct compromise of the pulmonary artery and proximal airways or pulmonary tumor embolism or indirect injury secondary to thromboembolism and inflammation [86]. Pulmonary hypertension was found in 25% of hematopoietic stem cell transplant patients with respiratory failure, likely from transplant-associated thrombotic microangiopathy, a severe small vessel angiopathy [87]. In addition, some cancer treatments have been associated with pulmonary hypertension. Bleomycin can cause interstitial lung disease and dasatinib, a second-generation tyrosine kinase inhibitor, has been associated with direct pulmonary vascular injury [86].

Pulmonary hypertension during pregnancy has long been considered to carry the highest risk of maternal and neonatal complications, especially maternal death, during labor and delivery and the immediate postpartum period [88]. Pregnant women with pulmonary hypertension experienced significantly higher major adverse cardiac events (24.8 versus 0.4%, *p* < 0.0001) compared to healthy women [88]. Although there are no data on pregnancy outcomes in cancer survivors with pulmonary hypertension, data suggest that patients with pulmonary hypertension of all etiologies, particularly pulmonary arterial hypertension, have a high risk of maternal mortality during or immediately after pregnancy.

### 5.8. Maternal Mortality

Cancer survivors have a higher maternal mortality compared with the general population. According to the National Inpatient Sample data of 64,506 pregnant cancer survivors, the survivors had significantly higher odds of death during delivery compared to pregnant women without a history of cancer (58 vs. 5 deaths per 100,000 pregnancies, *p* = 0.01) [69]. From the 2000–2016 Surveillance, Epidemiology, and End Results (SEER) registry of women with cancer aged 15 to 39 years, the maternal death rate was 10.4/100,000 person-years and the standard mortality ratio was 10.9 (95% CI, 9.36–12.7) compared with the general US population [89].

## 6. Preconception Counseling

Cancer therapies increase the maternal cardiovascular risk during pregnancy and labor. The Children’s Oncology Group (COG) considers the following women as at increased risk [90]:

- Women who had radiation to the pelvis, lower spine, or total body;
- Women who received anthracycline chemotherapy and women who received radiation to the abdomen, chest, or thoracic spine.

**Figure 2:**
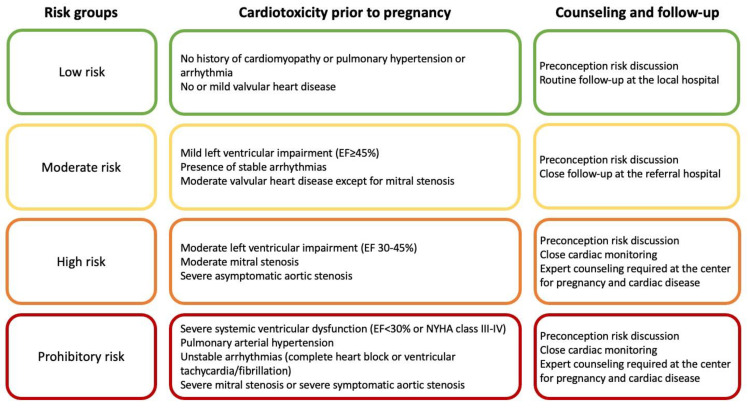
A proposed risk stratification and approach to counseling maternal cardiovascular risk in childhood, adolescent, and young adult cancer survivors. EF = ejection fraction; NYHA = New York Heart Association.

Preconception counseling is critical to evaluating and optimizing pregnancy-related risks in cancer survivors. Based on the modified World Health Organization (mWHO) classification [91], we stratify the childhood, adolescent, and young adult cancer survivors into four risk groups and recommend corresponding counseling strategies (Figure 2). Survivors with cardiovascular sequelae of prior cancer therapy (moderate, high, and prohibitory risk) should be cared for by an expert multidisciplinary team, including obstetrics, cardiology, anesthesia, and specialized nursing, among others, as supported by multiple society guidelines [91,92,93].

According to the International Late Effects of Childhood Cancer Guideline Harmonization Group (IGHG), cardiomyopathy surveillance with echocardiography is reasonable before pregnancy or in the first trimester for all female survivors treated with anthracyclines and chest radiation [35]. Based on the baseline evaluation, cardiomyopathy surveillance during pregnancy can be considered when clinically indicated, 1 month before and 1 month after delivery for survivors with low and moderate risk. For survivors with high risk or prohibitory risk, cardiac surveillance with an echocardiogram can be considered during each trimester and 1 month after delivery and when clinically indicated [94].

## 7. Management

The management of cardiovascular disease during pregnancy has been detailed in many guidelines [91,92,93]. In general, in cancer survivors with normal baseline cardiac evaluation (low risk), counseling cardiovascular disease risk during pregnancy and alerting signs or symptoms related to potential cardiovascular dysfunction and routine follow-up as described above are recommended. In cancer survivors with abnormal baseline evaluation (moderate to high risk), a careful discussion about the potential deterioration of cardiac dysfunction and management during pregnancy and around delivery should be conducted. Existing cardiovascular conditions should be optimized before conception. Volume status of survivors with baseline cardiomyopathy or pericardial or valvular disease should be carefully monitored and managed during pregnancy and immediately after delivery due to the hemodynamic changes.

Cancer survivors with prohibitory risk of pregnancy should be counseled against pregnancy. If an intervention is absolutely necessary (for example, in cases of severe mitral stenosis and aortic stenosis), the best time is after the 4th month in the second trimester when organogenesis is complete, the fetal thyroid is still inactive, and the uterine volume is still small [91].

## 8. Conclusions

Thus far, data are limited addressing the maternal cardiovascular risk and outcomes in childhood, adolescent, and young adult cancer survivors. Female cancer survivors have an overall higher risk of maternal cardiovascular outcomes compared to general pregnancy, such as HF, preeclampsia, and maternal mortality. In female cancer survivors with normal cardiac function before pregnancy, the occurrence of the new development of HF during pregnancy is low. In survivors with cardiotoxicity prior to pregnancy, the risk of HF during and immediately after pregnancy appears to be much higher. Data are especially lacking to support the practical, safe, and cost-effective cardiac surveillance strategies during pregnancy. More research on characterizing the risk of cardiovascular events associated with pregnancy in cancer survivors and assessing the management strategies in this growing population is warranted.

## Figures and Tables

**Table 1:** Cardiotoxic effects of selected cancer therapies.

Drug	Study	Toxic Dose Range	Cardiac Toxicity	Frequency of Occurrence ^a^
Doxorubicin	Chlebowski 1979^30^	>450 mg/m^2^	Left ventricular dysfunction	Common
Epirubicin	Tjuljandin 1990^31^	>900 mg/m^2^		Common
Idarubicin	Anderlini 1995^32^	150–290 mg/m^2^		Intermediate
Paclitaxel	Perez 1998^33^	Conventional dose	Left ventricular dysfunction	Intermediate
Docetaxel	Kenmotsu & Tanigawara 2015^34^			Intermediate
Cyclophosphamide	Gottdiener 1981,^35^ Goldberg 1986^36^	>100–120 mg/kg	Left ventricular dysfunction	Intermediate
Ifosfamide	Kandylis 1989,^37^ Tascilar 2007,^38^ Cancer Care Ontario^39^	>10 mg/m^2^		Uncommon
Capecitabine	Sentürk 2009^40^	Conventional dose	Cardiac ischemia	Intermediate
Fluorouracil	Sentürk 2009,^40^ Schimmel 2004,^41^ Chanan-Khan 2004^42^			Intermediate
Paclitaxel	Perez 1998^33^	Conventional dose	Cardiac ischemia	Uncommon
Docetaxel	Kenmotsu & Tanigawara 2015^34^			Intermediate
Trabectedin	Lebedinsky 2011^43^	Conventional dose	Cardiac ischemia	Intermediate
Arsenic trioxide	Brana & Taberno 2010^44^	Conventional dose	QTc prolongation	Common
Paclitaxel	Perez 1998^33^	Conventional dose	QTc prolongation	Uncommon

a Common indicates that more than 5% reported incidence; intermediate, between 1% and 5% reported incidence; uncommon, less than 1% reported incidence. Obtain permission from CA. Cancer J. Clin. 2016, 66, 309–325 [19].

## Data Availability

Not applicable.
